# 

**Published:** 1894-06

**Authors:** 


### NOTICES.

### The Kentucky State Dental Association.

The twenty-fourth annual meeting of the Kentucky State Dental Association will be held at the Louisville College of Dentistry, Chestnut street, Louisville, on Tuesday, June 19, 1894, continuing in session three days.

A very interesting programme has been arranged for this meeting, including papers and discussion on the following subjects: "Formaldehyd;" "Secret Nostrums—Their Effect on Patient and Profession;" "Dental Parasites"; "My Mistakes;" "Dentistry in South America;" "Are Professional Lies Justifiable?" "A Few Practical Points;" "It Is Amusing." Other papers have been promised by the following members: Dr. A. Wilkes Smith, of Richmond; Drs. E. C. Dunn, E. M. Kettig, H. B. Tileston and A. B. Weaver, all of Louisville.

The clinics will also be of a very important and instructive nature, and the Executive Committee will see that all new appliances and methods are properly brought before the meeting.

All reputable members of the dental profession are cordially invited to be present at this meeting. "Come and bring your friends; the association is always glad to welcome visitors."

The State Board of Dental Examiners will hold their meeting during the session for the transaction of such business as may come before them.

Arrangements have been made with the proprietors of "The Fifth Avenue Hotel" and "The Willard" by which delegates will receive reduced rates.

For further or special information address any member of the Executive Committee, or

Dr. J. H. Baldwin, Secretary,
615 W. Chestnut st., Louisville, Ky.

The Executive Committee take great pleasure in stating to members and visitors, that through the kindness of Col. C. P. Atmore, General Passenger Agent, L. & N. R. R., arrangements with lines terminating here for one and one-third fare, on certificate plan, have been secured. Purchase tickets to Louisville and procure certificate from railroad agent, showing regular ticket has been sold, etc. Dr. J. H. Baldwin, our Secretary, will certify as to attendance, and presentation of such certificate to agent at Louisville, within three days after adjournment, will secure special return ticket at one-third fare, to points in Kentucky.

B. OSCAR DOYLE,

Secretary Executive Committee.

June 2, 1894.

### Illinois State Dental Society.

CHICAGO, May 21, 1894.

At the thirtieth annual meeting of the Illinois State Dental Society held at Springfield, May 8 to 11, 1894. the following officers were elected for the ensuing year:

J. W. Cormany, Mt. Carroll, President; S. F. Duncan, Joliet, Vice-President; Louis Ottofy, Chicago, Secretary; W. A. Stevens, Chicago, Treasurer; Grafton Munroe, Springfield, Librarian. The next meeting will be held at Galesburg, May 14 to 17, 1895.

LOUIS OTTOFY, *Secretary*,
Masonic Temple,
Chicago.

### The Indiana State Dental Society.

The annual meeting of the Indiana State Dental Society will be held on Tuesday, June 26, 1894, at Lake Maxinkuckee, Indiana, and will continue in session three days.

The State Board of Dental Examiners will meet at the same time and place.

A cordial invitation is extended to members of the dental profession.

G. E. HUNT, Secretary,
Indianapolis.

### The American Dental Association.

The annual meeting of the American Dental Association will be held at Old Point Comfort on the first Tuesday of August next, and the indications are that there will be a very large meeting; that is a very attractive and pleasant place for the meeting.

The reports are that extensive preparation is being made by a large number of the membership for work at that meeting.

Persons going to the meeting either from or through Cincinnati, will find the Chesapeake & Ohio Railway by far the most desirable route, and this is the only direct way. The best train for the party to take is the Number 4, which leaves Cincinnati at 7 o'clock P. M. This is a solid vestibule train, and has a through Pullman sleeper from Cincinnati to Old Point Comfort, arriving there at 6 P. M. the following day. This train will give the party the benefit of the Alleghany and Blue Ridge Mountain scenery, Shenandoah and Piedmont Valleys in the day time, and on the return trip the corresponding train can be taken at Old Point, arriving at Cincinnati at 5 P. M. the following evening.

The fare for the round trip will be fifteen ($15.70) dollars and seventy cents. Arrangements have been made with hotels for reduced rates, so that the opportunity for a visit to this delightful place will be afforded at a very economical cost. Let every member go and take his family.

---

FOR the benefit of those who may be at a loss as to a form for a professional card the following, which came to hand a few days ago, is a veritable sample, with the name and location slightly changed:

"DR. JOHN BENNINGTON,

GENERAL-TOOTH-DENTIST,

Watchmaker-and-Jeweler.

GLEN ROVER, TENN."

### THE DENTAL REGISTER,

**A MONTHLY MAGAZINE DEVOTED TO THE INTERESTS OF THE**

### DENTAL PROFESSION.

*Terms Reduced to* $2.00 *Per Year. Single Copies,* 25 *Cents.*

**EDITED BY PROF. J. TAFT, D.D.S.**

—AND—

**Published by SAM'L A. CROCKER & CO., Ohio Dental and Surgical Depot.**

The volume commences in January and closes in December.

The REGISTER being issued monthly is always fresh, therefore Indispensable to the practitioner who desires to keep posted up with the new inventions and improvements which are daily developed. Neither expense nor effort will be spared to give value and variety to its contents. Articles will be illustrated with well executed engravings whenever they will add to the interest or assist to explanation.

Its extensive circulation and frequency of issue make it one of the BEST DENTAL ADVERTISING MEDIUMS in the United States.

All subscriptions must begin with January or July.

Local or special notices, five lines or less, will be inserted for $3 each insertion; over five lines, 50 cts. per line.

All advertising bills payable in advance, or at furthest, after the issue of the first number containing the advertisement. All transient advertisements to be paid for in advance.

☞CONTRIBUTIONS SOLICITED.

Exclusively Editorial Communications should be addressed to the Editor.

The latest number of the REGISTER may be ordered with or without other good at any time, and all letters should be addressed to

